# Two first-in-human studies of xentuzumab, a humanised insulin-like growth factor (IGF)-neutralising antibody, in patients with advanced solid tumours

**DOI:** 10.1038/s41416-020-0774-1

**Published:** 2020-03-12

**Authors:** Johann de Bono, Chia-Chi Lin, Li-Tzong Chen, Jesus Corral, Vasiliki Michalarea, Karim Rihawi, Michael Ong, Jih-Hsiang Lee, Chih-Hung Hsu, James Chih-Hsin Yang, Her-Shyong Shiah, Chia-Jui Yen, Alan Anthoney, Maria Jove, Susanne Buschke, René Fuertig, Ulrike Schmid, Rainer-Georg Goeldner, Natalja Strelkowa, Dennis Chin-Lun Huang, Thomas Bogenrieder, Chris Twelves, Ann-Lii Cheng

**Affiliations:** 1 Drug Development Unit, Royal Marsden Hospital & Institute of Cancer Research, Downs Road, Sutton, UK; 2 Department of Oncology, National Taiwan University Hospital, 7 Chung Shan S. Rd., Taipei, Taiwan; 3 Department of Internal Medicine, National Cheng Kung University Hospital, National Cheng Kung University, 138 Sheng Li Road, Tainan, Taiwan; 4 Department of Internal Medicine, Kaohsiung Medical University Hospital, Kaohsiung Medical University, 100 Tzyou 1st Road, Kaohsiung, Taiwan; 5 National Institute of Cancer Research, National Health Research Institutes, 367 Sheng Li Road, Tainan, Taiwan; 6 Medical Oncology Department, Clinica Universidad de Navarra, Calle Marquesado de Sta. Marta 1, Madrid, Spain; 7 Azienda Sanitaria Universitaria Integrata di Udine, Via Pozzuolo, 330, 33100 Udine, Italy; 8 The Ottawa Hospital Cancer Centre, 501 Smyth Road, Ottawa, ON Canada; 9 The Ph.D. Program for Cancer Biology and Drug Discovery, College of Medical Science and Technology, Taipei Medical University, 250 Wuxing Street, Taipei, Taiwan; 10 University of Leeds and Leeds Teaching Hospitals Trust, Beckett Street, Leeds, UK; 11 Translational Medicine & Clinical Pharmacology, Boehringer Ingelheim Pharma GmbH & Co. KG, Birkendorfer Str. 65, Biberach an der Riß, Germany; 12 Biostatistics and Data Sciences, Boehringer Ingelheim Pharma GmbH & Co KG, Birkendorfer Str. 65, Biberach an der Riß, Germany; 13 Medical Department, Boehringer Ingelheim Taiwan Ltd, 12F, No. 2, Sec 3, Minsheng East Road, Taipei, Taiwan; 14 Department of Urology, University Hospital Grosshadern, Ludwig-Maximilians-University, 1 Geschwister-Scholl-Platz, Munich, Germany; 15 Medicine and Translational Research, Boehringer Ingelheim RCV, 5-11 Doktor-Boehringer-Gasse, Vienna, Austria; 16 National Taiwan University Cancer Center, Taipei, Taiwan

**Keywords:** Cancer therapy, Oncogenes

## Abstract

**Background:**

Xentuzumab, an insulin-like growth factor (IGF)-1/IGF-2-neutralising antibody, binds IGF-1 and IGF-2, inhibiting their growth-promoting signalling. Two first-in-human trials assessed the maximum-tolerated/relevant biological dose (MTD/RBD), safety, pharmacokinetics, pharmacodynamics, and activity of xentuzumab in advanced/metastatic solid cancers.

**Methods:**

These phase 1, open-label trials comprised dose-finding (part I; 3 + 3 design) and expansion cohorts (part II; selected tumours; RBD [weekly dosing]). Primary endpoints were MTD/RBD.

**Results:**

Study 1280.1 involved 61 patients (part I: xentuzumab 10–1800 mg weekly, *n* = 48; part II: 1000 mg weekly, *n* = 13); study 1280.2, 64 patients (part I: 10–3600 mg three-weekly, *n* = 33; part II: 1000 mg weekly, *n* = 31). One dose-limiting toxicity occurred; the MTD was not reached for either schedule. Adverse events were generally grade 1/2, mostly gastrointestinal. Xentuzumab showed dose-proportional pharmacokinetics. Total plasma IGF-1 increased dose dependently, plateauing at ~1000 mg/week; at ≥450 mg/week, IGF bioactivity was almost undetectable. Two partial responses occurred (poorly differentiated nasopharyngeal carcinoma and peripheral primitive neuroectodermal tumour). Integration of biomarker and response data by Bayesian Logistic Regression Modeling (BLRM) confirmed the RBD.

**Conclusions:**

Xentuzumab was well tolerated; MTD was not reached. RBD was 1000 mg weekly, confirmed by BLRM. Xentuzumab showed preliminary anti-tumour activity.

**Clinical trial registration:**

NCT01403974; NCT01317420.

## Background

The insulin-like growth factor (IGF) signalling axis plays a role in carcinogenesis and is associated with cancer progression, prognosis, and treatment resistance.^1^ Consequently, therapeutic targeting of the IGF axis has been investigated in various human cancers, with early strategies targeting the IGF type 1 receptor (IGF-1R) using anti-IGF-1R monoclonal antibodies (mAbs) and IGF-1R tyrosine kinase inhibitors (TKIs).^2^ So far, there has been limited success with these agents, although this may reflect the lack of validated predictive biomarkers to allow patient enrichment.^3^

Xentuzumab (BI 836845) is a fully humanised IgG1 mAb, which binds IGF-1 and IGF-2 with high affinity and potently neutralises proliferative and pro-survival signalling triggered by both ligands.^4^ This ligand-binding approach offers advantages over IGF-1R-targeted therapies, as it inhibits the proliferative/anti-apoptotic effects of IGF-2 signalling through insulin receptor isoform A (INSR-A).^5^ Additionally, IGF-1/-2-neutralising mAbs have a lower potential for hyperglycaemia than IGF-1R/INSR TKIs, as they do not affect the metabolic INSR isoform B.^5^ In preclinical models, xentuzumab showed potent anti-proliferative effects against a range of cancer cell lines, and demonstrated encouraging anti-tumour activity and a favourable safety profile in vivo.^4^

Here, two first-in-human phase 1 studies were conducted in parallel to explore two dosing schedules (administration once weekly and every 3 weeks) to determine the maximum-tolerated dose (MTD) and/or relevant biological dose (RBD) of intravenous (IV) xentuzumab in patients with advanced solid cancers, considering safety, anti-tumour activity, and pharmacokinetics (PK)/pharmacodynamics (PD).

## Methods

### Study design and patients

Study 1280.1 (NCT01403974), conducted in Taiwan (August 2011 to June 2016), and study 1280.2 (NCT01317420), conducted in the UK (June 2011 to December 2015), were phase 1, open-label, dose-escalation trials, each comprising two parts. Part I was a dose-finding period and used a 3 + 3 dose-escalation design, while part II evaluated expansion cohorts of patients with selected tumour types. The primary objective of part I was to identify the MTD of xentuzumab (or the RBD in the absence of an MTD). To assess the optimal dosing schedule, xentuzumab was given once weekly in study 1280.1 and every 3 weeks in study 1280.2. The objectives of part II were to evaluate anti-tumour activity at the MTD/RBD, and to further evaluate the safety and PK/PD of xentuzumab.

Eligible patients (aged ≥ 18 years) had pathologically confirmed advanced/metastatic solid tumours and had failed or were not amenable to standard therapy. Patients were required to have evaluable disease or at least one measurable lesion per Response Evaluation Criteria in Solid Tumours version 1.1 (RECIST v1.1), an Eastern Cooperative Oncology Group performance status of 0, 1, or 2, and adequate haematological, hepatic, and renal function. Patients entering part II were required to have cytologically or histologically confirmed disease from the Ewing sarcoma family of tumours or peripheral primitive neuroectodermal tumours (pPNET; cohort 1) or solid tumours suitable for biopsy (cohort 2). Key exclusion criteria included active infectious disease, serious illness, or concomitant disease considered by the investigator to be incompatible with the protocol. Patients who had not recovered from toxicities related to previous anti-cancer therapy (to a severity of grade 1 or less; Common Terminology Criteria for Adverse Events version 4.03 [CTCAE v4.03]) were excluded, as were those with a history of diabetes mellitus or untreated/symptomatic brain metastases.

### Treatment

In part I, patients received xentuzumab by 1-h IV infusion, either weekly (study 1280.1; days 1, 8 and 15) or every 3 weeks (study 1280.2; day 1). The starting dose was 10 mg, and this dose was doubled until CTCAE grade ≥2 drug-related adverse events (AEs) occurred. Thereafter, dose escalations used incremental steps equivalent to 20–50% of the previously evaluated dose. In part II of both studies, patients were treated weekly at the MTD/RBD determined in part I. Treatment continued until disease progression, clinically unacceptable AEs, or other reasons necessitating withdrawal.

In the event of a dose-limiting toxicity (DLT), treatment could be delayed for up to 4 weeks, and supportive therapy continued or initiated (see Supplementary Methods for further information on DLT criteria). In patients with documented clinical benefit (stable disease [SD] or objective response [complete response (CR) or partial response (PR)]), xentuzumab could be resumed at a reduced dose level (maximum of two dose reductions), after events had recovered to the baseline severity or CTCAE grade 1.

### Endpoints and assessments

The primary endpoint in part I was the MTD/RBD. The MTD was defined as the highest dose level of xentuzumab at which no more than 1/6 patients experienced a DLT during the first 21-day cycle. If the MTD was not reached, an RBD could be determined using PK and biomarker data to infer target engagement; tumour assessment data could also be considered.

AEs were graded according to CTCAE v4.03. Serial blood samples were collected for determination of anti-drug antibodies (ADA) and to determine plasma levels of xentuzumab and PD biomarkers such as IGF-1, IGF-2, IGF binding protein 3 (IGFBP-3), and IGF-1R phosphorylation. Ex vivo IGF-1R phosphorylation in a cellular assay is considered a surrogate for IGF-1 and IGF-2 activity in patients’ plasma and will be referred to as ‘bioactive IGF’ throughout this manuscript.^6^ Plasma samples were analysed for ADA using a validated electrochemiluminescence method. Xentuzumab plasma concentrations were determined using a validated enzyme-linked immunosorbent assay (lower limit of quantification, 200 ng/mL). Tumour assessment was performed according to RECIST v1.1 at screening, every two cycles for the first six treatment cycles, and every three cycles thereafter. Disease control was defined as CR/PR or SD lasting ≥24 weeks. Analyses were descriptive and exploratory; no formal statistical tests were performed.

Exploratory Bayesian logistic regression models (BLRMs),^7,8^ were applied to confirm the RBD, using pooled data from all patients who received weekly xentuzumab. A binary response criterion (Yes/No) for each endpoint (saturation of total IGF-1 in plasma, inhibition of IGF-bioactivity, and disease control) was assessed per patient, to create input data for the BLRMs (Supplementary Methods).

## Results

### Patients and treatment

In total, 61 patients were treated in study 1280.1 (part I, *n* = 48; part II, *n* = 13) and 64 patients in study 1280.2 (part I, *n* = 33; part II, *n* = 31; Table 1; Supplementary Fig. S1). All patients in study 1280.1 were Asian and 97% of patients in study 1280.2 were white (3% were Asian). All patients discontinued treatment, most commonly due to progressive disease (study 1280.1, 77%; study 1280.2, 91%). In part I, patients received once-weekly xentuzumab at 14 dose levels from 10 to 1800 mg (study 1280.1), or every 3 weeks at 11 dose levels from 10 to 3600 mg (study 1280.2). During part II of both studies, patients received xentuzumab at the RBD (1000 mg once weekly), determined via an integrated analysis of safety, PK, biomarker and disease control data from both studies. Treatment exposure is shown in Table 2.

**Table 1:** Baseline characteristics.

	Study 1280.1 (weekly xentuzumab)^a^		
	Part I (*n* = 48)	Part II (*n* = 13)	Total (*N* = 61)
Male/female, *n* (%)	34 (71)/14 (29)	4 (31)/9 (69)	38 (62)/ 23 (38)
Median age, years (range)	57.5 (19–76)	58.0 (29–72)	58.0 (19–76)
Race, *n* (%)			
Asian	48 (100)	13 (100)	61 (100)
Black/African American	0	0	0
White	0	0	0
Baseline ECOG PS, *n* (%)			
0	24 (50)	5 (38)	29 (48)
1	22 (46)	8 (62)	30 (49)
2	2 (4)	0	2 (3)
Type of cancer, *n* (%)^b^			
Liver	7 (15)	1 (8)	8 (13)
Oesophagus	7 (15)	0	7 (11)
Colorectal	5 (10)	1 (8)	6 (10)
Soft tissue/osteosarcoma	3 (6)	2 (15)	5 (8)
Biliary tree	2 (4)	1 (8)	3 (5)
Endocrine cancers	3 (6)	0	3 (5)
Pleura	3 (6)	0	3 (5)
Thyroid and parathyroid	2 (4)	1 (8)	3 (5)
Endometrial cancer	0	2 (15)	2 (3)
Other	16 (33)	5 (38)	21 (34)
Prior anticancer therapy, *n* (%)			
Systemic chemotherapy	43 (90)	13 (100)	56 (92)
Surgery	40 (83)	9 (69)	49 (80)
Molecular targeted therapy	10 (21)	0	10 (16)
Hormone therapy	3 (6)	0	3 (5)
Immunotherapy	2 (4)	0	2 (3)
Biological therapy	0	0	0
Other	32 (67)	5 (38)	37 (61)

	Study 1280.2 (3-weekly xentuzumab)^a^		
	Part I (*n* = 33)	Part II (*n* = 31)	Total (*N* = 64)
Male/female, *n* (%)	20 (61)/13 (39)	20 (65)/11 (35)	40 (63)/24 (38)
Median age, years (range)	59.0 (23–79)	50.0 (19–77)	55.0 (19–79)
Race, *n* (%)			
Asian	2 (6)	0	2 (3)
Black/African American	0	0	0
White	31 (94)	31 (100)	62 (97)
Baseline ECOG PS, *n* (%)			
0	10 (30)	8 (26)	18 (28)
1	21 (64)	22 (71)	43 (67)
2	2 (6)	1 (3)	3 (5)
Type of cancer, *n* (%)^b^			
Colorectal	6 (18)	6 (19)	12 (19)
Soft tissue/osteosarcoma	0	11 (35)	11 (17)
Adrenal	4 (12)	0	4 (6)
Ovary	2 (6)	2 (6)	4 (6)
GI tract	1 (3)	2 (6)	3 (5)
Oesophagus	1 (3)	2 (6)	3 (5)
Head and neck cancers	2 (6)	1 (3)	3 (5)
Lung	1 (3)	1 (3)	2 (3)
Mesothelial cancers	1 (3)	1 (3)	2 (3)
NSCLC	2 (6)	0	2 (3)
Pancreas	2 (6)	0	2 (3)
Prostate	2 (6)	0	2 (3)
Other	9 (27)	5 (16)	14 (22)
Prior anticancer therapy, *n* (%)			
Systemic chemotherapy	31 (94)	31 (100)	62 (97)
Surgery	23 (70)	24 (77)	47 (73)
Hormone therapy	4 (12)	1 (3)	5 (8)
Molecular targeted therapy	4 (12)	0	4 (6)
Immunotherapy	1 (3)	0	1 (2)
Biological therapy	0	1 (3)	1 (2)
Other	8 (24)	13 (42)	21 (33)

^a^In part I, all doses (all patients in part II received xentuzumab 1000 mg weekly).

^b^Cancer type present in at least two patients in either part of the study.

*ECOG PS* Eastern Cooperative Oncology Group performance status, *GI* gastrointestinal, *NSCLC* non-small-cell lung cancer.

**Table 2:** Summary of exposure, overall safety summary and most common drug-related AEs (occurring in >2 patients in either study).

	Study 1280.1 (weekly xentuzumab)^a^			Study 1280.2 (3-weekly xentuzumab)^a^		
Patients, *n* (%)	Part I (*n* = 48)	Part II (*n* = 13)	Total (*N* = 61)	Part I (*n* = 33)	Part II (*n* = 31)	Total (*N* = 64)
Exposure to xentuzumab treatment						
Treatment duration, days, median (range)	43 (1–282)	78 (1–498)	57 (1–498)	22 (1–232)	36 (1–162)	26 (1–232)
Sum of treatment duration, years	10.2	5.0	15.2	3.7	4.4	8.1
Number of infusions, median (range)	6 (1–40)	12 (1–71)	9 (1–71)	2 (1–11)	6 (1–22)	N/A
Overall AE summary						
Any AE	46 (96)	12 (92)	58 (95)	33 (100)	31 (100)	64 (100)
Highest CTCAE grade AE						
Grade 3	10 (21)	3 (23)	13 (21)	14 (42)	9 (29)	23 (36)
Grade 4	4 (8)	1 (8)	5 (8)	2 (6)	1 (3)	3 (5)
Grade 5	3 (6)	0	3 (5)	0	0	0
DLT^b^	1 (2)	0	1 (2)	0	0	0
Drug-related AE	10 (21)	2 (15)	12 (20)	14 (42)	15 (48)	29 (45)
AE leading to discontinuation	5 (10)	0	5 (8)	1 (3)	2 (6)	3 (5)
AE leading to dose reduction	0	0	0	0	0	0
Any SAE	17 (35)	4 (31)	21 (34)	9 (27)	15 (48)	24 (38)
Drug-related AEs, *n* (%)						
Fatigue	0	0	0	3 (9)	4 (13)	7 (11)
Nausea	1 (2)	0	1 (2)	4 (12)	3 (10)	7 (11)
Lethargy	0	0	0	5 (15)	1 (3)	6 (9)
Decreased appetite	0	0	0	3 (9)	2 (6)	5 (8)
Diarrhoea	0	0	0	2 (6)	3 (10)	5 (8)
Constipation	0	0	0	3 (9)	0	3 (5)
Infusion-related reaction	0	0	0	0	3 (10)	3 (5)
Vomiting	1 (2)	1 (8)	2 (3)	0	1 (3)	1 (2)
Hyperglycaemia	0	0	0	1 (3)	1 (3)	2 (3)
Lymphocyte count decreased	2 (4)	0	2 (3)	0	0	0
Platelet count decreased	2 (4)	0	2 (3)	0	0	0
White blood cell count decreased	2 (4)	0	2 (3)	0	0	0
Anaemia	1 (2)	1 (8)	2 (3)	0	0	0
Neutropenia	0	0	0	0	2 (6)	2 (3)
Thrombocytopenia	0	0	0	0	2 (6)	2 (3)
Oral candidiasis	0	0	0	2 (6)	0	2 (3)

^a^In part I, all doses (all patients in part II received xentuzumab 1000 mg weekly).

^b^Grade 3 pulmonary haemorrhage due to bleeding from a vessel adjacent to tumour in 1 patient treated with xentuzumab 450 mg weekly.

*AE* adverse event, *CTCAE* Common Terminology Criteria for Adverse Events, *DLT* dose-limiting toxicity, *N/A* not applicable, *SAE* serious adverse event.

### DLTs and MTD

Only one DLT was observed (grade 3 pulmonary haemorrhage due to bleeding from a vessel adjacent to the tumour in a patient with follicular thyroid cancer [study 1280.1; xentuzumab 450 mg/week; Table 2]). In study 1280.1, dose escalation reached 1800 mg/week without additional DLTs. No DLTs occurred with xentuzumab given every 3 weeks (range 10–3600 mg); consequently, the MTD was not reached with either schedule. In the absence of an MTD, the preliminary RBD (1000 mg) was determined by combining data from both phase 1 studies. An exploratory BLRM was conducted to confirm the RBD (see below for further details).

### Safety and tolerability

An overall summary of AEs and most common drug-related AEs for xentuzumab given once weekly (study 1280.1) and every 3 weeks (study 1280.2) is shown in Table 2. The most common AEs, regardless of causality, were those pertaining to gastrointestinal disorders (Supplementary Table S1). Most AEs were mild (CTCAE grade 1/2). Grade ≥3 AEs occurred in 17 (part I) and 4 patients (part II) in study 1280.1, and in 16 (part I) and 10 patients (part II) in study 1280.2 (Table 2). The most common drug-related AE across both studies was nausea (mostly grade 1/2; study 1280.1: part I, one patient; study 1280.2: part I, four patients; part II, three patients; Table 2).

In study 1280.1, serious AEs (SAEs) were reported in 21 patients (34%); only one was considered to be related to the study drug (grade 3 pulmonary haemorrhage resulting in discontinuation; also identified as a DLT). Twenty-four patients (38%) in study 1280.2 had a SAE; in five, these were considered to be drug-related, as follows: grade 2 infusion-related reaction (*n* = 2), grade 3 infusion-related reaction, grade 3 hyperglycaemia, and grade 2 hypersensitivity (each *n* = 1).

Five patients (8%) in study 1280.1 discontinued due to the following AEs: pneumonia, metastases to the central nervous system, pneumonia aspiration, pulmonary haemorrhage, and subdural haemorrhage (all in part I). Three patients (5%) in study 1280.2 discontinued due to the following AEs: pneumonia (part I), *clostridium difficile* colitis and infusion-related reaction (both part II). Only grade 3 pulmonary haemorrhage (study 1280.1) and grade 2 infusion-related reaction (study 1280.2) were considered to be drug-related. Three patients had AEs that resulted in death (acute respiratory failure [40 mg weekly dose], dyspnoea [60 mg weekly dose], and malignant neoplasm progression [1800 mg weekly dose]); all occurred in study 1280.1, part I, but none were considered to be drug-related. The patients who had acute respiratory failure and dyspnoea both had lung metastases at the start of treatment and these AEs were associated with progression of their disease.

### Immunogenicity

The immunogenic reaction to xentuzumab was low in both studies. In study 1280.1, 5/235 ADA samples during part I and 0/103 ADA samples during part II had confirmed ADA-positive results; however, most samples in part I and 85/103 samples in part II were ADA-inconclusive due to low drug tolerance limits. Overall, only 4/60 patients could reliably be determined as ADA-negative, whereas 53 patients had to be defined as ADA-negative but inconclusive. In study 1280.2, 7/115 ADA samples in part I, and 1/108 ADA samples in part II had confirmed ADA-positive results. However, 12/115 samples in part I and 72/108 samples in part II were ADA-inconclusive. Overall, only 15/64 patients could reliably be determined as ADA-negative, whereas 43 patients had to be defined as ADA-negative but inconclusive. ADA-positive and ADA-inconclusive patients did not exhibit obvious differences in their PK and PD profiles compared with confirmed ADA-negative patients (data not shown).

### Pharmacokinetics/pharmacodynamics

**Fig. 1:**
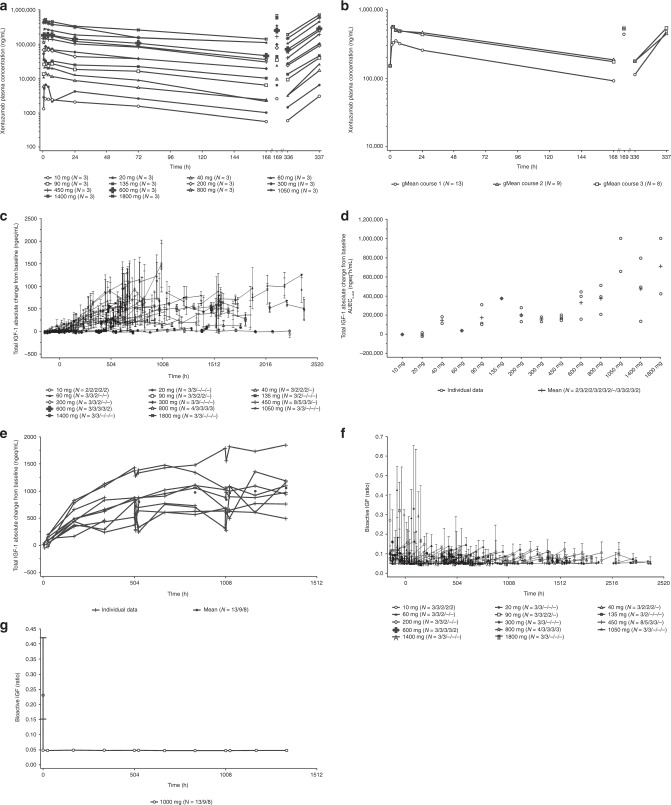
PK and PD effects of xentuzumab in study 1280.1 (weekly xentuzumab). Mean plasma concentration–time profiles after IV infusion of xentuzumab in part I course 1 (**a**; semi-log scale), and after the first (course 1) and repeated (course 2 and 3) weekly IV infusions of 1000 mg xentuzumab in part II (**b**; semi-log scale). Mean total IGF-1 absolute change from baseline–time profiles after repeated weekly xentuzumab infusions in part I (**c**). Comparison of individual and arithmetic mean AUEC_0-840_ values of absolute change from baseline of total IGF-1 after weekly xentuzumab infusions of 10–1800 mg (**d**). Individual and arithmetic mean total IGF-1 absolute change from baseline–time profiles after weekly IV infusion of 1000 mg in part II (**e**; linear scale; filled circles indicate the mean values). Median bioactive IGF effect–time profiles after repeated weekly infusions of xentuzumab in part I (**f**), and after weekly IV infusion of 1000 mg in part II (**g**; linear scale). *AUEC*_*0–840*_ area under the biomarker effect versus time curve between 0 and 840 h after the start of the first infusion, *gMean* geometric mean, *IGF(-1)* insulin-like growth factor(-1), *IV* intravenous, *PD* pharmacodynamics, *PK* pharmacokinetics.

Non-compartmental PK parameters are summarised in Table 3. Xentuzumab exhibited at least biphasic disposition kinetics in both studies; after reaching the maximum, concentrations declined rapidly during the initial 24-h period, and more slowly thereafter. Exposure increased in proportion to the dose over both dose ranges tested (10–1800 mg weekly; 10–3600 mg every 3 weeks; Fig. 1a, Supplementary Fig. S2a). The geometric mean terminal half-life across all dose groups was 6.7 days (range 5.2–9.1). Steady-state conditions were achieved after approximately 4–5 weeks in part I of both studies (weekly infusions: 31 days [range 9.5–51]; infusions every 3 weeks: 37 days). Weekly dosing led to an accumulation ratio of approximately 1.5, based on *C*_max_ and AUC_0–168_ values, whereas no accumulation was detected after repeated dosing every 3 weeks (study 1280.2, part I). Geometric mean plasma concentration–time profiles in part II (1000 mg/week) were similar between the two studies (Fig. 1b, Supplementary Fig. S2b).

**Table 3:** Non-compartmental PK parameters.

Part I	Study 1280.1, dose-escalation part (weekly dosing)						Study 1280.2, dose-escalation part (3-weekly dosing)						
		PK parameters in course 1, gMean (gCV%)						PK parameters in course 1, gMean (gCV%)					
Dose, mg	Max *n*	AUC_0–168_, µg h/mL	AUC_0–168,norm_, µg h/mL/mg	*C*_max_, µg/mL	*C*_max,norm_, µg/mL/mg	*t*_max_,^a^ *h*	Max *n*	AUC_0–504_, µg h/mL	AUC_0–504,norm_, µg h/mL/mg	*C*_max_, µg/mL	*C*_max,norm_, µg/mL/mg	*t*_max_,^a^ *h*	*t*_1/2,_ *h*
10	3	249 (37.2)	24.9 (37.2)	2.87 (23.8)	0.287	2.10 (1.15–4.00)	3	363 (1.79)	36.3 (1.79)	2.45 (10.6)	0.245 (10.6)	2.17 (0.93–4.00)	183 (7.82)
20	3	390 (32.9)	19.5 (32.9)	6.53 (14.9)	0.326	2.02 (1.75–6.38)	3	724 (26.8)	36.2 (26.8)	5.24 (46.1)	0.262 (46.1)	2.00 (1.28–2.07)	154 (26.1)
40	3	752 (66.3)	18.8 (66.3)	13.8 (26.6)	0.346	2.03 (2.00–2.03)	3	993 (30.9)	24.8 (30.9)	10.7 (65.8)	0.268 (65.8)	1.00 (1.00–2.05)	161 (31.4)
60	3	1300 (42.5)	21.7 (42.5)	22.2 (3.78)	0.370	1.13 (1.03–1.92)	0	–	–	–	–	–	–
80^b^	0	–	–	–	–	–	2^b^	2510 (8.20)	31.3 (8.20)	19.5 (9.80)	0.244 (9.80)	3.00 (2.08–3.92)	156 (9.53)
90	3	2250 (16.7)	25.0 (16.7)	28.2 (28.7)	0.313	2.00 (1.80–7.00)	0	–	–	–	–	–	–
135	3	3050 (17.7)	22.6 (17.7)	36.6 (20.6)	0.271	1.05 (1.03–1.92)	0	–	–	–	–	–	–
160	0	–	–	–	–	–	3	5270 (21.6)	33.0 (21.6)	66.2 (113)	0.414 (113)	1.00 (0.50–2.00)	148 (10.9)
200	3	5950 (26.9)	29.7 (26.9)	70.8 (26.7)	0.354	2.00 (2.00–3.77)	0	–	–	–	–	–	–
300	3	5680 (23.1)	18.9 (23.1)	81.9 (42.2)	0.273	2.25 (2.07–2.28)	0	–	–	–	–	–	–
320	0	–	–	–	–	–	3	12,100 (27.6)	37.9 (27.6)	196 (163)	0.612 (163)	2.00 (1.00–2.00)	203 (27.6)
450	8	10,600 (12.6)	23.6 (12.6)	151 (16.7)	0.336	1.18 (1.02–7.00)	0	–	–	–	–	–	–
600	3	15,600 (7.09)	26.0 (7.09)	199 (10.8)	0.332	2.00 (1.92–2.25)	0	–	–	–	–	–	–
640	0	–	–	–	–	–	3	17,300 (61.7)	27.0 (61.7)	146 (47.8)	0.228 (47.8)	2.00 (2.00–4.00)	145 (29.5)
800	4	14,500 (45.6)	18.1 (45.6)	200 (38.1)	0.250	1.25 (1.07–2.00)	0	–	–	–	–	–	–
1050	3	31,100 (64.9)	29.6 (64.9)	270 (38.9)	0.257	1.17 (1.02–4.02)	0	–	–	–	–	–	–
1280	0	–	–	–	–	–	3	34,600 (68.2)	27.0 (68.2)	421 (30.8)	0.329 (30.8)	2.28 (2.00–4.00)	129 (5.08)
1400	3	31,800^c^	22.7^c^	415 (13.0)	0.296	2.00 (1.00–2.00)	0	–	–	–	–	–	–
1800	3	36,200^c^	20.1^c^	503 (13.4)	0.279	2.00 (1.00–2.00)	3	42,100 (6.55)	23.4 (6.55)	727 (101)	0.404 (101)	2.00 (0.93–2.00)	150 (5.50)
2400	0	–	–	–	–	–	3	78,500 (6.32)	32.7 (6.32)	554 (11.5)	0.231 (11.5)	4.00 (2.27–6.73)	218 (20.2)
3600	0	–	–	–	–	–	3	87,800 (24.9)	24.4 (24.9)	1080 (10.3)	0.299 (10.3)	1.82 (1.82–3.00)	124 (19.2)

Part II	Study 1280.1, expansion part (1000 mg weekly)						Study 1280.2, expansion part (1000 mg weekly)						
		PK parameters, gMean (gCV%)						PK parameters, gMean (gCV%)					
Course	Max *n*	AUC_0–168_, µg h/mL	AUC_0–168,norm_, µg h/mL/mg	*C*_max_, µg/mL	*C*_max,norm_, µg/mL/mg	*t*_max_,^a^ *h*	Max *n*	AUC_0–168_, µg h/mL	AUC_0–168,norm_, µg h/mL/mg	*C*_max_, µg/mL	*C*_max,norm_, µg/mL/mg	*t*_max_,^a^ *h*	
1	13	32,200 (27.4)	32.2 (27.4)	386 (30.7)	0.386 (30.7)	4.00 (1.00–7.00)	30	24,100 (22.1)	24.1 (22.1)	295 (19.9)	0.295 (19.9)	2.51 (0.92–7.00)	
2	9	55,500 (20.4)	55.5 (20.4)	607 (10.4)	0.607 (10.4)	2.00 (1.00–24.00)	21	37,300 (33.3)	37.3 (33.3)	399 (26.7)	0.399 (26.7)	2.98 (0.92–7.02)	
3	8	53,700 (25.4)	53.7 (25.4)	616 (24.8)	0.616 (24.8)	1.50 (0.98–7.00)	11	36,900 (41.6)	36.9 (41.6)	419 (26.5)	0.419 (26.5)	1.03 (0.92–4.92)	

^a^Median (range).

^b^Excluding one patient with an invalid sampling time point and no sample at 504 h.

^c^Individual values.

*AUC*_*0–168*_ area under the plasma concentration–time curve over the time interval of 1 week, *AUC*_*0–504*_ area under the plasma concentration–time curve over the time interval of 3 weeks, *C*_*max*_ maximum plasma concentration, *gCV* geometric coefficient of variation, *gMean* geometric mean, *max* maximum, *norm* normalised, *h* hours, *PK* pharmacokinetics, *T*_*max*_ time from dosing to maximum plasma concentration, *t*_*1/2*_ terminal half-life.

Total IGF-1, total IGF-2, total IGFBP-3 and bioactive IGF in plasma were measured as markers of target engagement. Total IGF-1 concentrations increased dose dependently in both studies. With weekly dosing, levels accumulated after repeated administration (study 1280.1, part I; Fig. 1c), reaching a plateau at doses of ≥1050 mg/week (Fig. 1c, d). No plateau was reached by any of the investigated doses administered every 3 weeks (Supplementary Fig. S2c). The mean absolute change from baseline–time profile of total IGF-1 in part II (1000 mg/week) was a similar shape and time course in both studies, reaching a plateau after 4–5 weeks (Fig. 1e, Supplementary Fig. S2d).

In both studies, IGF bioactivity declined rapidly after the start of the xentuzumab infusion (Fig. 1f, Supplementary Fig. S2e). At doses of ≥450 mg/week, the median IGF bioactivity remained at the limit of detection (LOD) for the entire dosing interval (Fig. 1f, g, Supplementary Fig. S2f), while doses of up to 3600 mg given every 3 weeks did not reduce bioactive IGF to the same extent (Supplementary Fig. S2e).

No clear dose- or time-dependent effects of xentuzumab on total IGF-2 and IGFBP-3 were detected in either study.

### Anti-tumour activity

In part I of study 1280.1, two patients who received xentuzumab once weekly at doses close to the RBD achieved durable PRs (Table 4). The first (poorly differentiated nasopharyngeal carcinoma; 800 mg) had a PR lasting 20.7 weeks (censored), and the second (pPNET; 1050 mg) had a PR lasting 30.4 weeks (censored); both responses were detected first at the initial post-treatment assessment, on days 42 and 41, respectively. Three patients in part I of study 1280.1 achieved durable SD (≥24 weeks; urothelial carcinoma [10 mg], malignant mesothelioma [10 mg], and cardiac adenocarcinoma [600 mg]), as did three patients in part II of study 1280.1 (all 1000 mg; endometrial carcinoma, retroperitoneal leiomyosarcoma, and thymic carcinoma). There were no objective responses in study 1280.2 (xentuzumab every 3 weeks); however, two patients in part I of study 1280.2 (cervical adenocarcinoma [320 mg] and prostate adenocarcinoma [2400 mg]) achieved durable SD.

**Table 4:** Best overall response.

	Study 1280.1 (weekly xentuzumab)^a^			Study 1280.2 (3-weekly xentuzumab)^a^		
Patients, *n* (%)	Part I (*n* = 48)	Part II (*n* = 13)	Total (*N* = 61)	Part I (*n* = 33)	Part II (*n* = 31)	Total (*N* = 64)
Best overall response						
CR	0	0	0	0	0	0
PR	2 (4)	0	2 (3)	0	0	0
SD^b^	22 (46)	8 (62)	30 (48)	10 (30)	13 (42)	23 (36)
SD lasting ≥ 24 weeks	3 (6)	3 (23)	6 (10)	2 (6)	0	2 (3)
Progressive disease	20 (42)	3 (23)	23 (38)	21 (64)	11 (35)	32 (50)
NE	4 (8)	2 (15)	6 (13)	2 (6)	7 (23)	9 (14)

^a^In part I (all patients in part II received xentuzumab 1000 mg weekly).

^b^Meeting the minimum time of 35 days from first xentuzumab infusion.

*CR* complete response, *NE* not evaluable, *PR* partial response, *SD* stable disease.

### Integration of biomarker and efficacy data (BLRM)

**Fig. 2:**
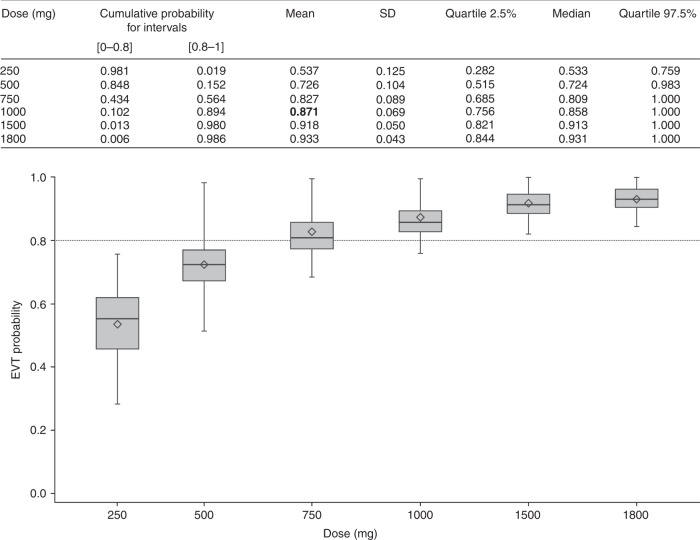
Probability that RBD is reached for weekly dose levels (pooled data from both studies). Mean probability that RBD is reached for xentuzumab 1000 mg/week is shown in bold. Horizontal lines represent the median, diamonds represent the mean, and boxes represent the 25th and 75th percentiles. Whiskers represent the 2.5th and 97.5th percentiles. *EVT* extreme value theory, *RBD* relevant biological dose.

At the end of the study, all three endpoints for the BLRMs indicated a dose-dependent increase of positive events, reaching a plateau at around 1000 mg, i.e. the probability of reaching the RBD accumulated at around 1000 mg (Supplementary Table S2). The clearest signal was obtained from the saturation of total IGF-1 in plasma. Integrating all the data, the estimated posterior probability of having reached the RBD at each weekly dose level is shown in Fig. 2. The RBD was considered to have been reached if the posterior probability was >80%; at 1000 mg, this was 87%. The RBD was confirmed to be 1000 mg weekly and was taken into further clinical investigation in part II of studies 1280.1 and 1280.2, and in new combination studies.

## Discussion

These two first-in-human trials established the RBD for xentuzumab as 1000 mg weekly IV; the MTD was not reached. The RBD was determined based on an integrated analysis of available data from both studies, including safety, PK, biomarker data, and disease control. In study 1280.2, the 3-weekly schedule did not show a similar level of target engagement at equivalent doses to the weekly schedule; as such, xentuzumab 1000 mg weekly was assessed in the expansion part in both trials. At the end of the trial, data integration performed using BLRMs was found to support the previously determined RBD.

The safety profile of xentuzumab was clinically manageable and tolerable, with only one DLT observed across both studies. AEs were generally mild-to-moderate in intensity, the most common pertaining to gastrointestinal disorders. Hyperglycaemia is a known class effect of anti-IGF-1R mAbs and TKIs, despite their lack of interference with insulin binding.^1^ The finding that drug-related hyperglycaemia was rare with xentuzumab (no patients in study 1280.1 and two patients in study 1280.2 [grade 1 and grade 3]) is consistent with clinical studies of another IGF-ligand blocking mAb, dusigitumab (MEDI-573).^9,10^ The low incidence of hyperglycaemia with xentuzumab suggests that the anti-ligand mechanism of action may be more favourable than IGF-1R-targeted therapies with regard to hyperglycaemia. The immunogenic reaction to xentuzumab was low; however, no firm conclusions on ADA incidence and prevalence could be made in either study due to a high frequency of samples being deemed ADA inconclusive.

Xentuzumab showed dose-proportional plasma PK, with a mean terminal half-life of 6.7 days, together with attainment of steady state after 4–5 weeks. Accordingly, accumulation was moderate after weekly infusions. No obvious differences in xentuzumab PK were observed between the two phase 1 trials conducted in Europe and Asia (geometric mean dose normalised parameters AUC_0–168,norm_ and *C*_max,norm_ from course 1, part II of study 1280.2 [1000 mg/week dose] were within the range of corresponding values from different weekly doses in study 1280.1).

Consistent with preclinical studies,^11^ increases in total IGF-1 and a reduction in bioactive IGF in plasma demonstrated indirect and direct target engagement, respectively. The increase in total IGF-1 is assumed to result from two mechanisms: (1) a reduced elimination of IGF-1 following binding to xentuzumab; and (2) a growth-hormone-dependent negative feedback mechanism via the pituitary, which may increase IGF-1 secretion by the liver. The plateau in total IGF-1 observed at 1050 mg/week is hypothesised to indicate that, at this dose, most IGF-1 in plasma is bound to xentuzumab, and that the growth-hormone-dependent feedback mechanism regulating IGF-1 secretion is saturated. No plateau in total IGF-1 increase was reached with the investigated doses given every 3 weeks. A reduction in IGF bioactivity to the LOD throughout the dosing interval was observed with weekly doses ≥450 mg. Infusions given every 3 weeks, even at the highest dose, did not reduce bioactive IGF to the same extent. While xentuzumab also neutralises IGF-2, no clear effects of xentuzumab on total IGF-2 were detected in these phase 1 studies. We hypothesise that this may be due to a lower binding affinity of xentuzumab to IGF-2 than IGF-1, as well as a higher binding affinity of IGF-2 to IGFBPs than IGF-1. IGF-2 synthesis is also not affected by the growth-hormone-dependent feedback mechanism. Nevertheless, there is evidence to suggest that free (bioactive) IGF-2 was reduced through binding to xentuzumab. Direct measurements of IGF-2 were not possible in the clinic; however, bioactive IGF levels (surrogate for both IGF-1 and IGF-2 activity) decreased during xentuzumab treatment. Furthermore, the effect of xentuzumab on free IGF-1 and IGF-2 was assessed in a separate mechanistic PK-PD modelling study describing the dynamics and interactions of IGF-1, IGF-2, and IGFBPs in the absence and presence of xentuzumab.^12^ This quantitative framework, developed by combining published in vitro and in vivo information with clinical data from the xentuzumab phase 1 studies, enabled prediction of the concentrations of free IGF-1 and IGF-2. Simulations indicated a high neutralisation of free IGF-1 and IGF-2 over time (by at least 91% and 64%, respectively; steady state versus baseline) at a xentuzumab dose of 1000 mg/week.^12^

Xentuzumab showed preliminary anti-tumour activity in pre-treated patients with advanced solid tumours, with two PRs observed at doses close to the RBD (once weekly dosing) and durable SD (≥24 weeks) reported in eight patients. These findings are consistent with two phase 1 studies of dusigitumab, which reported SD in Caucasian (US) and Asian (Japanese) patients.^9,10^

The results of the BLRM, integrating biomarkers (plateau in total IGF-1; inhibition of IGF bioactivity), and efficacy data from patients in both studies, provided further confirmation of the RBD. In the BLRM model, the estimated probability of having reached the RBD with a dose of 1000 mg was 87%, indicating that saturation of total IGF-1 biomarker, inhibition of IGF bioactivity, and disease control would plateau around this dose.

While targeting of the IGF-1R pathway has been challenging, with several failures in the clinical setting,^13^ we consider that the distinct mechanism of action of xentuzumab (neutralisation of IGF ligands) may offer advantages versus those previously employed with IGF-1R mAbs and IGF-1R TKIs. Indeed, xentuzumab was associated with a low incidence of hyperglycaemia compared with anti-IGF-1R mAbs and TKIs. The favourable safety profile of xentuzumab also offers the potential of combination with other cancer therapies. Given the involvement of IGF signalling in resistance to other anti-cancer therapies,^1^ this is a key area of interest. It is also anticipated that inhibition of both IGF-1 and IGF-2 may offer efficacy benefits over IGF-1R-targeted treatments by also inhibiting proliferative signalling via IGF-2 activation of INSR-A, but this remains to be determined in larger scale trials. Identification of potential biomarkers will also be important to identify patient subgroups who may gain particular benefit.

In conclusion, the RBD for xentuzumab was determined to be 1000 mg/week and was recommended as the dose for further clinical evaluation. Clinical development of xentuzumab in combination with other anti-cancer therapies is feasible based on its favourable safety profile and biological activity in these studies. The safety and anti-tumour activity of xentuzumab, as well as potential biomarkers, is being assessed further in breast and prostate cancers.

## Supplementary information

Supplementary Material

## Data Availability

To ensure independent interpretation of clinical study results, Boehringer Ingelheim grants all external authors access to all relevant material, including participant-level clinical study data, and relevant material as needed by them to fulfil their role and obligations as authors under the ICMJE criteria. Furthermore, clinical study documents (e.g. study report, study protocol, statistical analysis plan) and participant clinical study data are available to be shared after publication of the primary paper in a peer-reviewed journal and if regulatory activities are complete and other criteria met per the BI Policy on Transparency and Publication of Clinical Study Data: https://trials.boehringer-ingelheim.com/transparency_policy.html. Prior to providing access, documents will be examined, and, if necessary, redacted and the data will be de-identified, to protect the personal data of study participants and personnel, and to respect the boundaries of the informed consent of the study participants. Clinical Study Reports and Related Clinical Documents can be requested via this link: https://trials.boehringer-ingelheim.com/trial_results/clinical_submission_documents.html. All such requests will be governed by a Document Sharing Agreement. Bona fide, qualified scientific and medical researchers may request access to de-identified, analysable participant clinical study data with corresponding documentation describing the structure and content of the datasets. Upon approval, and governed by a Data Sharing Agreement, data are shared in a secured data-access system for a limited period of 1 year, which may be extended upon request. Researchers should use https://trials.boehringer-ingelheim.com to request access to study data.
