# Supplementary material for: Two first-in-human studies of xentuzumab, a humanised insulin-like growth factor (IGF)-neutralising antibody, in patients with advanced solid tumours
Source: Br J Cancer. 2020 Mar 12;122(9):1324–32. doi: 10.1038/s41416-020-0774-1 (PMC7188670; doi:10.1038/s41416-020-0774-1)
Supplement: Supplementary file 1 — Supplementary Material [file 41416_2020_774_MOESM1_ESM.docx]

**Supplementary Methods**

Definition of DLT

- A dose limiting toxicity (DLT) was defined as a drug-related adverse event (AE) with any of the following criteria:
  - Grade 4 neutropenia (< 0.5 x 10^9^/L) lasting ≥ 7 days;
  - Febrile neutropenia with an absolute neutrophil count (ANC) of < 1.0 x 10^9^/L with a single temperature of ≥ 38.3°C or a sustained temperature of ≥ 38°C for more than 1 h or a documented infection with ANC of < 1.0 x 10^9^/L;
  - Grade 4 thrombocytopenia (< 25.0 x 10^9^/L) or grade 3 thrombocytopenia associated with bleeding requiring platelet transfusion;
  - Grade ≥ 3 increased hepatic aspartate aminotransferase (AST) or alanine aminotransferase (ALT) > 5 to 20.0 x the upper limit of normal (ULN);
  - Grade 3/4 non-haematologic toxicity (except alopecia, incompletely treated nausea, untreated vomiting, untreated diarrhoea, skin toxicity, fatigue, infusion reaction, electrolyte, hyperglycaemia, AST or ALT);
  - Grade ≥ 2 infusion reaction despite adequate pre-medication;
  - Grade ≥ 2 nausea and/or vomiting persisting for 7 or more days despite antiemetic treatment;
  - Grade ≥ 3 skin toxicity despite adequate supportive care measures for up to 2 weeks if it did not reach an improvement to grade ≤ 2;
  - Grade ≥ 3 hyperglycaemia resistant to treatment with anti-diabetic agents;
  - Grade 3 electrolyte AE refractory to optimal correction therapy;
  - Non-recovery from a non-DLT grade > 2 toxicity to grade 1 within 14 days;
  - Sustained grade 3 fatigue or asthenia for longer than 96 h associated with deterioration of Eastern Cooperative Oncology Group performance score;
  - Any other study drug related toxicity considered significant enough to be qualified as a DLT in the opinion of any of the investigators and confirmed by the safety review with the Boehringer Ingelheim clinical monitor was reported as a DLT.

Description of the Bayesian Logistic Regression Model

The logistic regression model is defined as follows:

*pEff* is the probability of having the efficacy signal disease control = Yes when giving dose *d* of xentuzumab. *pIGF* is the probability that the biomarker total insulin-like growth factor (IGF)-1 has reached the saturation in a patient at dose *d*, and *pBioact* is the probability that inhibition of IGF bioactivity has been observed at dose *d*.

Logistic regression was used to model the relationship between the endpoints and the dose of xentuzumab for each endpoint separately:

- Efficacy – disease control Yes/No: logit(*pEff*) =log(*α_1_*) + *β_1_*log(*d/d**)
- Biomarker – total IGF-1 saturation Yes/No: logit(*pIGF*) =log(*α_2_*) + *β_2_*log(*d/d**)
- Biomarker – inhibition of IGF bioactivity Yes/No: logit(*pBioact*) =log(*α_3_*) + *β_3_*log(*d/d**)

where logit(p)=log(p/(1-p)), *d** denotes the reference dose, and θ_i_ = (log(α_i_), log(β_i_)) with α_i_, β_i_ > 0, i=1,2,3 is the parameter vector of the respective model. Consequently, we have in total a 6-parametric model.

Here, the dose *d** = 500 mg was used as the reference dose for xentuzumab. Under independence of the three endpoints (disease control, total IGF-1 saturation, and inhibition of IGF bioactivity), the probability of having reached the relevant biological dose (RBD) in at least one endpoint while administering the dose *d* is:

$$pTDI(d)= pIGF(d) + pEff(d) + pBioact(d) - pIGF(d)*pEff(d) - pEff(d)*pBioact(d) - pIGF(d)*pBioact(d) + pIGF(d)*pBioact(d)*pEff(d)$$

This is a union of probabilities under independency assumption with corresponding odds:

$$odds(pTDI(d)) = pTDI(d)/(1- pTDI(d)).$$

The likelihood is then:

$$r(d) \sim Binomial(n(d), pTDI(d)).$$

In order to explore a potential positive (higher than expected under independence) or negative (lower than expected under independence) interaction between the three endpoints, a sensitivity analysis was performed on the expanded model containing all terms from above and an additional interaction term:

$$odds(pTD(d)) = odds(pTDI(d)) exp(\eta(d/d*)^2)$$

where -∞<*η*<∞ and *pTD*(*d*) is used in the likelihood instead of *pTDI*:

$$r(d) \sim Binomial(n(d), pTD).$$

Since a Bayesian approach was applied, prior distributions *f*(.) for each of the parameter vectors *θ_1_=(log(α_1_),log(β_1_)), θ_2_=(log(α_2_),log(β_2_)), θ_3_=(log(α_3_),log(β_3_))* and for the interaction term *η* needed to be specified.

The prior distributions for *θ_k_* (*k*=1,2,3) were specified as a mixture of three bivariate normal distributions:

$$f \left( \theta_{k} \right)= a_{1k} f_{1} (\theta_{k}) + a_{2k} f_{2} \left( \theta_{k} \right)+ a_{3k} f_{3} (\theta_{k})$$

with *a_1k_, a_2k_, a_3k_* the prior mixture weights (*a_1k_* + *a_2k_* + *a_3k_* = 1) and *f_i_*(*θ_k_*) = MVN(*μ_ik_*, *Σ_ik_*) (*i*=1,2) a bivariate normal distribution with mean vector μ_ik_ and covariance matrix Σ_ik_ where:

$$\Sigma_{ik}=\left( \begin{matrix} {\sigma^{2}}_{ik,11} & \sigma_{ik,11}\sigma_{ik,22}\rho_{ik} \\ \sigma_{ik,11}\sigma_{ik,22}\rho_{ik} & {\sigma^{2}}_{ik,22} \end{matrix} \right)$$

The estimated probability of having reached the RBD at each dose level from the model was reported as mean and standard deviation and additionally as cumulative probability in the following intervals:

Under dosing: [0.00, 0.8]

Targeted dosing: [0.8, 1]

The RBD was considered to have been reached if the posterior probability of the true target dose probability in the target interval [0.8, 1] was above 80%.

**Supplementary Fig. S1** Patient disposition. **
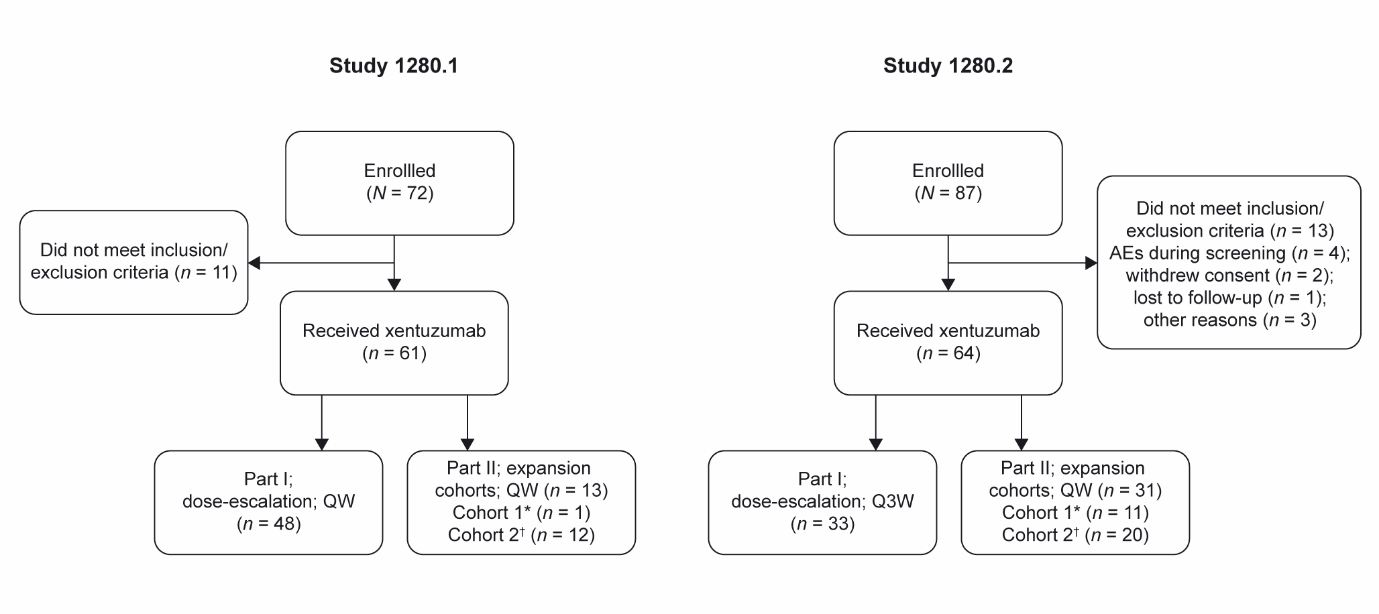
**

*Cytologically or histologically confirmed disease from the Ewing family of tumours or primitive neuroectodermal tumours. ^†^Solid tumours suitable for biopsy. *AE* adverse event, *QW* weekly, *Q3W* once every 3 weeks

**Supplementary Fig. S2** PK and PD effects of xentuzumab in study 1280.2 (3-weekly xentuzumab).

**
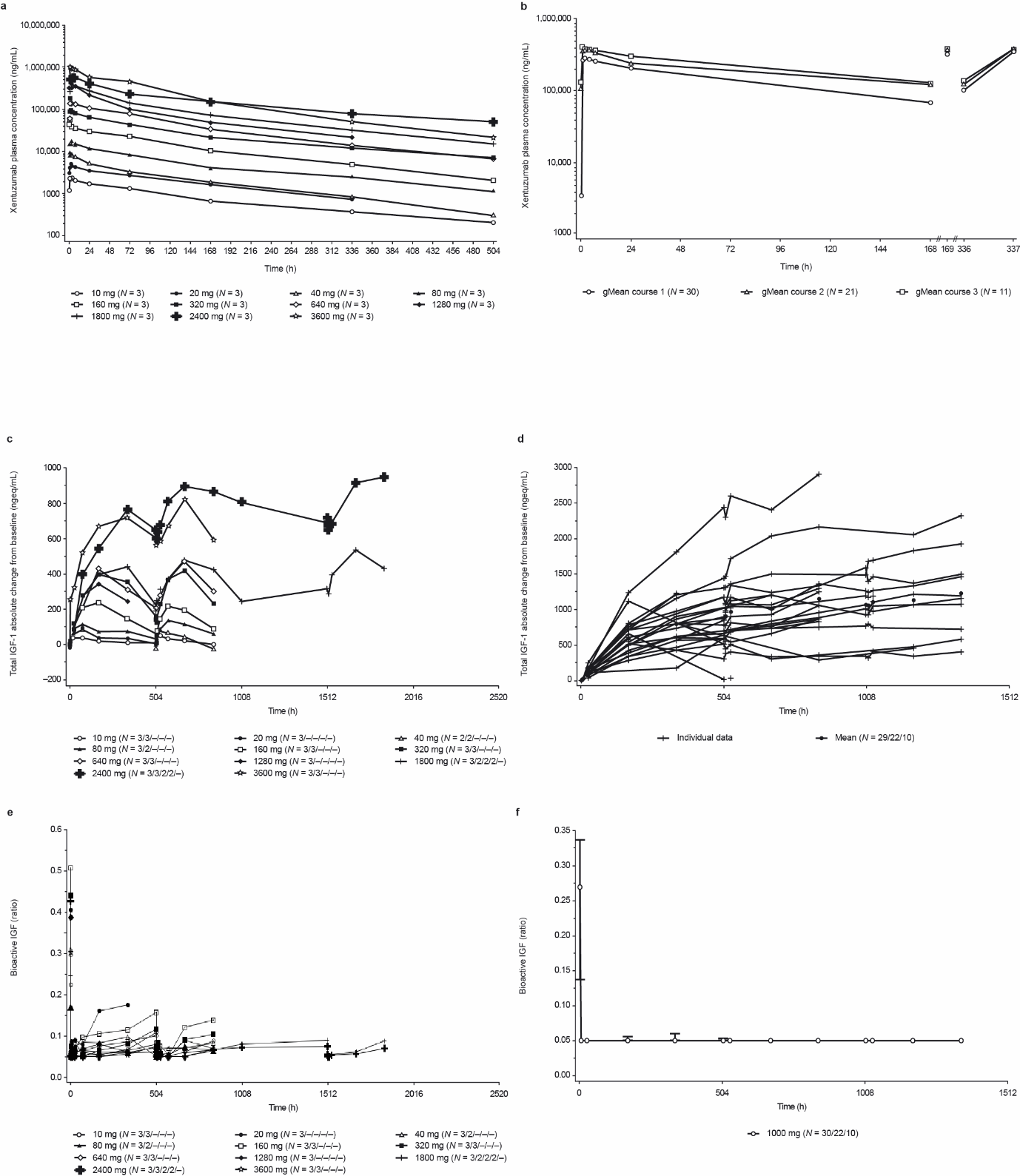
**

gMean plasma concentration–time profiles after IV infusion of xentuzumab in part I course 1 (**a**), and after the first (course 1) and repeated (courses 2 and 3) weekly IV infusions of 1000 mg xentuzumab in part II (**b**; semi-log scale). Mean total IGF-1 absolute change from baseline–time profiles after repeated three-weekly infusions of xentuzumab in part I (**c**), and individual and arithmetic mean total IGF-1 absolute change from baseline–time profiles after weekly IV infusion of 1000 mg in part II (**d**; linear scale; filled circles indicate the mean values). Median bioactive IGF effect–time profiles after repeated 3-weekly infusions of xentuzumab in part I (**e**), and after weekly IV infusion of 1000 mg in part II (**f**). *gMean* geometric mean, *IGF* insulin-like growth factor, *IV* intravenous, *PK* pharmacokinetic, *PD* pharmacodynamics

| **Supplementary Table S1.** Adverse events occurring in either study | | | | | | | | |
| --- | --- | --- | --- | --- | --- | --- | --- | --- |
|  | Study 1280.1 (weekly xentuzumab) | | | | | | | |
|  | Part I (*n* = 48) | | | | Part II (*n* = 13) | | | |
| SOC/preferred term, *n* (%) | All grades | Grade 3 | Grade 4 | Grade 5 | All grades | Grade 3 | Grade 4 | Grade 5 |
| Gastrointestinal disorders | 28 (58) | 1 (2) | 1 (2) | 0 | 10 (77) | 1 (8) | 0 | 0 |
| Abdominal pain | 2 (4) | 0 | 1 (2) | 0 | 0 | 0 | 0 | 0 |
| Gastrointestinal mucosal  disorder | 1 (2) | 0 | 1 (2) | 0 | 0 | 0 | 0 | 0 |
| Dysphagia | 3 (6) | 1 (2) | 0 | 0 | 1 (8) | 1 (8) | 0 | 0 |
| Respiratory, thoracic and  mediastinal disorders | 25 (52) | 1 (2) | 2 (4) | 2 (4) | 4 (31) | 1 (8) | 0 | 0 |
| Dyspnoea | 8 (17) | 2 (4) | 0 | 1 (2) | 1 (8) | 1 (8) | 0 | 0 |
| Pneumonia aspiration | 2 (4) | 0 | 1 (2) | 0 | 0 | 0 | 0 | 0 |
| Productive cough | 4 (8) | 1 (2) | 0 | 0 | 0 | 0 | 0 | 0 |
| Acute respiratory failure | 1 (2) | 0 | 0 | 1 (2) | 0 | 0 | 0 | 0 |
| Pulmonary haemorrhage | 1 (2) | 1 (2) | 0 | 0 | 0 | 0 | 0 | 0 |
| Respiratory failure | 1 (2) | 0 | 1 (2) | 0 | 0 | 0 | 0 | 0 |
| General disorders and  administrative site conditions | 21 (44) | 2 (4) | 0 | 0 | 7 (54) | 1 (8) | 0 | 0 |
| Chest pain | 7 (15) | 1 (2) | 0 | 0 | 1 (8) | 0 | 0 | 0 |
| Oedema | 1 (2) | 1 (2) | 0 | 0 | 0 | 0 | 0 | 0 |
| Pain | 1 (2) | 0 | 0 | 0 | 1 (8) | 1 (8) | 0 | 0 |
| Musculoskeletal and  connective tissue disorders | 17 (35) | 0 | 0 | 0 | 7 (54) | 2 (15) | 0 | 0 |
| Neck pain | 0 | 0 | 0 | 0 | 1 (8) | 1 (8) | 0 | 0 |
| Pain in extremity | 3 (6) | 0 | 0 | 0 | 2 (15) | 1 (8) | 0 | 0 |
| Investigations | 21 (44) | 2 (4) | 0 | 0 | 3 (23) | 1 (8) | 0 | 0 |
| Weight decreased | 11 (23) | 1 (2) | 0 | 0 | 3 (23) | 0 | 0 | 0 |
| Lymphocyte count  decreased | 3 (6) | 1 (2) | 0 | 0 | 0 | 0 | 0 | 0 |
| AST increased | 2 (4) | 0 | 0 | 0 | 1 (8) | 1 (8) | 0 | 0 |
| Metabolism and nutrition  disorders | 17 (35) | 1 (2) | 0 | 0 | 5 (38) | 0 | 0 | 0 |
| Hyponatremia | 1 (2) | 1 (2) | 0 | 0 | 0 | 0 | 0 | 0 |
| Nervous system disorders | 15 (31) | 3 (6) | 0 | 0 | 3 (23) | 1 (8) | 0 | 0 |
| Cerebral ischaemia | 1 (2) | 1 (2) | 0 | 0 | 0 | 0 | 0 | 0 |
| Facial paresis | 1 (2) | 1 (2) | 0 | 0 | 0 | 0 | 0 | 0 |
| Syncope | 1 (2) | 1 (2) | 0 | 0 | 0 | 0 | 0 | 0 |
| Cauda equina syndrome | 0 | 0 | 0 | 0 | 1 (8) | 1 (8) | 0 | 0 |
| Infections and infestations | 13 (27) | 3 (6) | 2 (4) | 0 | 4 (31) | 1 (8) | 0 | 0 |
| Pneumonia | 3 (6) | 2 (4) | 0 | 0 | 0 | 0 | 0 | 0 |
| Liver abscess | 2 (4) | 0 | 1 (2) | 0 | 0 | 0 | 0 | 0 |
| Device-related infection | 1 (2) | 1 (2) | 0 | 0 | 0 | 0 | 0 | 0 |
| Infection | 1 (2) | 1 (2) | 0 | 0 | 0 | 0 | 0 | 0 |
| Septic shock | 1 (2) | 0 | 1 (2) | 0 | 0 | 0 | 0 | 0 |
| Bacteraemia | 0 | 0 | 0 | 0 | 1 (8) | 1 (8) | 0 | 0 |
| Blood and lymphatic system disorders | 10 (21) | 4 (8) | 0 | 0 | 2 (15) | 1 (8) | 0 | 0 |
| Anaemia | 8 (17) | 3 (6) | 0 | 0 | 2 (15) | 1 (8) | 0 | 0 |
| Febrile neutropenia | 1 (2) | 1 (2) | 0 | 0 | 0 | 0 | 0 | 0 |
| Renal and urinary disorders | 9 (19) | 1 (2) | 0 | 0 | 2 (15) | 0 | 0 | 0 |
| Urinary retention | 3 (6) | 1 (2) | 0 | 0 | 0 | 0 | 0 | 0 |
| Hydronephrosis | 1 (2) | 1 (2) | 0 | 0 | 0 | 0 | 0 | 0 |
| Vascular disorders | 3 (6) | 2 (4) | 0 | 0 | 2 (15) | 0 | 1 (8) | 0 |
| Hypertension | 1 (2) | 1 (2) | 0 | 0 | 1 (8) | 0 | 0 | 0 |
| Hypotension | 1 (2) | 1 (2) | 0 | 0 | 0 | 0 | 0 | 0 |
| Superior vena cava syndrome | 0 | 0 | 0 | 0 | 1 (8) | 0 | 1 (8) | 0 |
| Injury, poisoning and  procedural complications | 3 (6) | 0 | 1 (2) | 0 | 2 (15) | 0 | 0 | 0 |
| Subdural haemorrhage | 1 (2) | 0 | 1 (2) | 0 | 0 | 0 | 0 | 0 |
| Hepatobiliary disorders | 5 (10) | 1 (2) | 1 (2) | 0 | 0 | 0 | 0 | 0 |
| Hyperbilirubinaemia | 2 (4) | 0 | 1 (2) | 0 | 0 | 0 | 0 | 0 |
| Jaundice cholestatic | 1 (2) | 1 (2) | 0 | 0 | 0 | 0 | 0 | 0 |
| Neoplasms benign,  malignant and unspecified  (including cysts and polyps) | 3 (6) | 1 (2) | 0 | 1 (2) | 1 (8) | 0 | 0 | 0 |
| Metastases to CNS | 2 (4) | 1 (2) | 0 | 0 | 0 | 0 | 0 | 0 |
| Malignant neoplasm progression | 1 (2) | 0 | 0 | 1 (2) | 0 | 0 | 0 | 0 |
| Endocrine disorders | 1 (2) | 0 | 1 (2) | 0 | 0 | 0 | 0 | 0 |
| Diabetes insipidus | 1 (2) | 0 | 1 (2) | 0 | 0 | 0 | 0 | 0 |
|  |  | | | | | | | |
|  | Study 1280.2 (three-weekly xentuzumab^a^) | | | | | | | |
|  | Part I (*n* = 33) | | | | Part II (*n* = 31) | | | |
| SOC/preferred term, *n* (%) | All grades | Grade 3 | Grade 4 | Grade 5 | All grades | Grade 3 | Grade 4 | Grade 5 |
| Gastrointestinal disorders | 25 (76) | 4 (12) | 0 | 0 | 23 (74) | 2 (7) | 0 | 0 |
| Diarrhoea | 11 (33) | 1 (3) | 0 | 0 | 5 (16) | 0 | 0 | 0 |
| Vomiting | 9 (27) | 1 (3) | 0 | 0 | 7 (23) | 1 (3) | 0 | 0 |
| Abdominal pain | 7 (21) | 1 (3) | 0 | 0 | 3 (10) | 0 | 0 | 0 |
| Oesophageal haemorrhage | 1 (3) | 1 (3) | 0 | 0 | 0 | 0 | 0 | 0 |
| Ascites | 0 | 0 | 0 | 0 | 1 (3) | 1 (3) | 0 | 0 |
| General disorders and  administrative site conditions | 17 (52) | 2 (6) | 0 | 0 | 17 (55) | 0 | 0 | 0 |
| Fatigue | 12 (36) | 2 (6) | 0 | 0 | 15 (48) | 0 | 0 | 0 |
| Metabolism and nutrition  disorders | 17 (52) | 2 (6) | 0 | 0 | 14 (45) | 2 (6) | 0 | 0 |
| Hypokalaemia | 6 (18) | 0 | 0 | 0 | 3 (10) | 1 (3) | 0 | 0 |
| Hypoalbuminaemia | 0 | 0 | 0 | 0 | 3 (10) | 1 (3) | 0 | 0 |
| Hypoglycaemia | 1 (3) | 1 (3) | 0 | 0 | 2 (6) | 0 | 0 | 0 |
| Hyponatremia | 1 (3) | 1 (3) | 0 | 0 | 1 (3) | 0 | 0 | 0 |
| Respiratory, thoracic and  mediastinal disorders | 11 (33) | 0 | 0 | 0 | 15 (48) | 1 (3) | 0 | 0 |
| Pleural effusion | 0 | 0 | 0 | 0 | 1 (3) | 1 (3) | 0 | 0 |
| Nervous system disorders | 14 (42) | 1 (3) | 0 | 0 | 6 (19) | 0 | 0 | 0 |
| Loss of consciousness | 1 (3) | 1 (3) | 0 | 0 | 0 | 0 | 0 | 0 |
| Musculoskeletal and  connective tissue disorders | 12 (36) | 3 (9) | 0 | 0 | 12 (39) | 0 | 0 | 0 |
| Back pain | 5 (15) | 3 (9) | 0 | 0 | 6 (19) | 0 | 0 | 0 |
| Infections and infestations | 12 (36) | 5 (15) | 2 (6) | 0 | 11 (35) | 4 (13) | 0 | 0 |
| UTI | 3 (9) | 0 | 0 | 0 | 5 (16) | 1 (3) | 0 | 0 |
| Lower respiratory tract infection | 2 (6) | 1 (3) | 0 | 0 | 3 (10) | 1 (3) | 0 | 0 |
| Aspergillus infection | 0 | 0 | 0 | 0 | 1 (3) | 1 (3) | 0 | 0 |
| *Clostridium difficile* colitis | 0 | 0 | 0 | 0 | 1 (3) | 1 (3) | 0 | 0 |
| Pseudomonas infection | 0 | 0 | 0 | 0 | 1 (3) | 1 (3) | 0 | 0 |
| Skin infection | 0 | 0 | 0 | 0 | 1 (3) | 1 (3) | 0 | 0 |
| Device-related infection | 1 (3) | 1 (3) | 0 | 0 | 0 | 0 | 0 | 0 |
| Escherichia UTI | 1 (3) | 1 (3) | 0 | 0 | 0 | 0 | 0 | 0 |
| Gastroenteritis norovirus | 1 (3) | 1 (3) | 0 | 0 | 0 | 0 | 0 | 0 |
| Pneumonia | 1 (3) | 0 | 1 (3) | 0 | 0 | 0 | 0 | 0 |
| Sepsis | 1 (3) | 0 | 1 (3) | 0 | 0 | 0 | 0 | 0 |
| Tooth abscess | 1 (3) | 1 (3) | 0 | 0 | 0 | 0 | 0 | 0 |
| Blood and lymphatic system disorders | 6 (18) | 0 | 0 | 0 | 11 (35) | 1 (3) | 1 (3) | 0 |
| Anaemia | 6 (18) | 0 | 0 | 0 | 8 (26) | 1 (3) | 0 | 0 |
| Thrombocytopenia | 0 | 0 | 0 | 0 | 4 (13) | 0 | 1 (3) | 0 |
| Injury, poisoning and  procedural complications | 1 (3) | 0 | 0 | 0 | 8 (26) | 1 (3) | 0 | 0 |
| Infusion-related reaction | 0 | 0 | 0 | 0 | 4 (13) | 1 (3) | 0 | 0 |
| Vascular disorders | 3 (9) | 3 (9) | 0 | 0 | 3 (10) | 0 | 0 | 0 |
| Hypertension | 3 (9) | 3 (9) | 0 | 0 | 0 | 0 | 0 | 0 |
| Hypotension | 1 (3) | 1 (3) | 0 | 0 | 1 (3) | 0 | 0 | 0 |
| Hepatobiliary disorders | 1 (3) | 1 (3) | 0 | 0 | 3 (10) | 0 | 0 | 0 |
| Hepatic function abnormal | 1 (3) | 1 (3) | 0 | 0 | 0 | 0 | 0 | 0 |
| Reproductive system and  breast disorders | 3 (9) | 0 | 0 | 0 | 2 (6) | 1 (3) | 0 | 0 |
| Vaginal haemorrhage | 1 (3) | 0 | 0 | 0 | 2 (6) | 1 (3) | 0 | 0 |
| Endocrine disorders | 1 (3) | 1 (3) | 0 | 0 | 1 (3) | 0 | 0 | 0 |
| Cushingoid | 1 (3) | 1 (3) | 0 | 0 | 1 (3) | 0 | 0 | 0 |
|  |  |  |  |  |  |  |  |  |
| ^a^In part I (all patients in part II received xentuzumab 1000 mg weekly)  *AST* aspartate aminotransferase, *CNS* central nervous system, *SOC* system organ class, *UTI* urinary tract infection | | | | | | | | |

| **Supplementary Table S2.** Achievement of binary criteria (Yes/No) for BLRM variables in patients receiving xentuzumab at different weekly dose levels (pooled data from weekly dosing of both studies) | | | | |
| --- | --- | --- | --- | --- |
|  |  | Primary endpoints for BLRM | | |
| Dose, mg | BLRM set, *n* | Patients with observed total IGF-1 saturation,^a^ *n* | Patients with observed IGF bioactivity inhibition,^b^ *n* | Patients with observed disease control,^c^ *n* |
| 10 | 3 | 0 | 0 | 2 |
| 20 | 3 | 0 | 1 | 0 |
| 40 | 3 | 0 | 0 | 0 |
| 60 | 3 | 0 | 0 | 0 |
| 90 | 3 | 1 | 1 | 0 |
| 135 | 3 | 2 | 1 | 0 |
| 200 | 3 | 2 | 0 | 0 |
| 300 | 3 | 1 | 1 | 0 |
| 450 | 8 | 0 | 3 | 0 |
| 600 | 3 | 2 | 2 | 1 |
| 800 | 4 | 3 | 2 | 1 |
| **1000** | **44** | **27** | **24** | **3** |
| 1050 | 3 | 3 | 2 | 1 |
| 1400 | 3 | 2 | 1 | 0 |
| 1800 | 3 | 3 | 1 | 0 |
|  | | | | |
| ^a^Saturation of total IGF-1 biomarker Yes/No: dose-dependent saturation of total IGF-1 with xentuzumab was inspected at the population level and determined to be Yes if the individual AUEC_0-672h_ of a patient was above the 90% lower CI  ^b^Inhibition of IGF-1R phosphorylation (IGF bioactivity) Yes/No: the result was considered Yes if the baseline measurement was above the LOD and the second pre-dose measurement in cycle 2 was equal to or below the LOD, i.e. a reduction was observed  ^c^Disease control Yes/No: disease control was defined as a best overall response according to RECIST 1.1 of CR, PR, or confirmed SD. To meet the definition of disease control with SD, the  patient had to have SD for ≥ 24 weeks  *AUEC_0–672h_* area under the effect curve from baseline to week 4, *BLRM* Bayesian logistic regression model, *CI* confidence interval, *CR* complete response, *IGF* insulin-like growth factor, *LOD* limit of detection, *PR* partial response, *RECIST,* Response Evaluation Criteria in Solid Tumors, *SD* stable disease | | | | |
